# Supplementary material for: Resolving the full spectrum of human genome variation using Linked-Reads
Source: Genome Res. 2019 Apr;29(4):635–45. doi: 10.1101/gr.234443.118 (PMC6442396; doi:10.1101/gr.234443.118)
Supplement: Supplemental Material [file supp_gr.234443.118_Supplemental_longranger-2.2.zip › longranger-2.2.tar.bz2!/lr2.2-public/lib/stan/cmdstan/stan/doc/api/html/json__error_8hpp_source.html]

Stan: src/stan/io/json/json\_error.hpp Source File


|  |  |
| --- | --- |
|  | Stan  2.5.0  probability, sampling & optimization |


- Main Page
- Related Pages
- Namespaces
- Classes
- Files

- File List
- File Members

All Classes Namespaces Files Functions Variables Typedefs Enumerations Enumerator Friends Macros Pages

- src
- stan
- io
- json

json\_error.hpp

Go to the documentation of this file.

1 #ifndef STAN\_\_IO\_\_JSON\_\_JSON\_ERROR\_HPP

2 #define STAN\_\_IO\_\_JSON\_\_JSON\_ERROR\_HPP

3

4 #include <stdexcept>

5

6

7 namespace stan {

8

9  namespace json {

10

14  struct json\_error : public std::logic\_error {

19  json\_error(const std::string& what)

20  : logic\_error(what) {

21  }

22

23  };

24

25

26  }

27 }

28 #endif

stan::json::json\_error::json\_error

json\_error(const std::string &what)

Construct a JSON error with the specified message.

**Definition:** json\_error.hpp:19

stan::json::json\_error

Exception type for JSON errors.

**Definition:** json\_error.hpp:14


---

[ Stan Home Page ]

*© 2011–2014,
Stan Development Team.*
